# Structure and Implications of JAMM, a Novel Metalloprotease

**DOI:** 10.1371/journal.pbio.0020017

**Published:** 2003-11-24

Proteins may be the workhorse of the cell, but when a cell can synthesize one protein in a matter of minutes, chances are some will become obsolete. Though many proteins put in years of productive service, others quickly outlive their usefulness and can even damage the cell. Proteins that help form bone and muscle, for example, function for years while regulators of mitosis and cell proliferation might finish their jobs in seconds. Such short-timers are soon tagged as superfluous by a chain of small proteins called ubiquitin, which marks the proteins for degradation in an enzyme called the proteasome. Once in the proteasome, these proteins are broken down and can then be recycled for more productive ventures.

A massive structure by cellular standards, the proteasome consists of multiple subunits, including a cylindrical core particle called 20S, which catalyzes degradation, and regulatory complexes called 19S caps, which form lid and base structures at both ends of the core. While the structure and biomechanics of the 20S core have been well characterized, much less is known about the functional mechanics of the regulatory complexes. The lid--base complex recognizes only ubiquitin-tagged proteins, which are then unfolded so they can enter the proteasome. But first ubiquitin chains must be detached from the protein, a task performed by an enzyme in the proteasome called Rpn11 isopeptidase. How the lid–base complex removes the ubiquitin tag, unfolds the protein, and shuttles it into the proteasome's core is not clear. Now Raymond Deshaies and colleagues present the structure of a homolog of the 19S lid's isopeptidase enzymatic center and provide new insights into these questions.

The proteasome Rpn11 subunit contains a key region called the JAMM motif, which Deshaies' lab has shown previously is required for the proteasome to remove ubiquitin tags. For the work discussed in this paper, the researchers set out to understand how the proteasome strips off ubiquitin tags from proteins about to be destroyed by determining the three-dimensional structure of the JAMM motif.

The researchers tested many genes to look for a JAMM-containing protein that would crystallize properly and found one in the heat-loving prokaryote Archaeoglobus fulgidus. After determining the structure of the JAMM protein (called AfJAMM), the researchers discovered that AfJAMM looks nothing like the well-known deubiquitinating enzymes. But the arrangement of a set of amino acids that binds a zinc ion and forms the proposed active site of AfJAMM does resemble that found in a well-known protein-degrading metalloprotease called thermolysin, even though in other respects AfJAMM and thermolysin have very different features. The researchers mutated amino acid residues in another JAMM protein called Csn5 (they expected these residues to be critical for isopeptidase activity as well, based on comparisons of the AfJAMM and thermolysin structures) and found that the residues are indeed important for Csn5 function. These results suggest that JAMM does indeed represent a novel family of metalloproteases.

As for the wider function of JAMM proteins, the researchers speculate that these proteins are likely to be involved in a variety of important regulatory systems since they appear in life forms that lack ubiquitin and ubiquitin-like proteins. The crystal structure reported in this paper will provide a valuable tool for investigations into the underlying structural and functional mechanisms of these enzymes. And it may have important therapeutic implications. Proteasome inhibitors are promising anticancer therapies—fighting cancer by blocking machinery required by rapidly dividing cells. In the hopes of developing more targeted therapies, scientists are trying to fine-tune their control of the ubiquitin system and the proteasome. Inhibiting the JAMM domain of enzymes like Csn5, which remove ubiquitin-like tags from proteins upstream of the proteasome, for example, might just do the trick.

**Figure:**
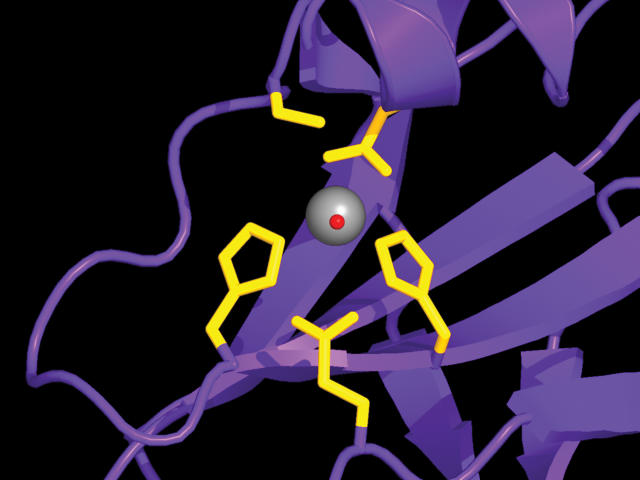
The active site of JAMM

